# Effect of all-trans retinoic acid on the proliferation and differentiation of brain tumor stem cells

**DOI:** 10.1186/1756-9966-29-113

**Published:** 2010-08-17

**Authors:** Chao Shi Niu, Ming Wu Li, Yong Feng Ni, Jian Min Chen, Jia Ming Mei, Jing Li, Xian Ming Fu

**Affiliations:** 1 Department of Neurosurgery, Anhui Provincial Hospital Affiliated to Anhui Medical University, Hefei, Anhui Province,230001, China; 2 Molecular Neurobiology & Neural Regeneration and Repairing Laboratory, Anhui Provincial Stereotactic Neurosurgical Institute, Hefei, Anhui Province, 230001, China

## Abstract

**Objective:**

To investigate the effect of all-trans retinoic acid(ATRA) on the proliferation and differentiation of brain tumor stem cells(BTSCs) *in vitro*.

**Methods:**

Limiting dilution and clonogenic assay were used to isolate and screen BTSCs from the fresh specimen of human brain glioblastoma. The obtained BTSCs, which were cultured in serum-free medium, were classified into four groups in accordance with the composition of the different treatments. The proliferation of the BTSCs was evaluated by MTT assay. The BTSCs were induced to differentiate in serum-containing medium, and classified into the ATRA group and control group. On the 10^th ^day of induction, the expressions of CD133 and glial fibrillary acidic protein (GFAP) in the differentiated BTSCs were detected by immunofluorescence. The differentiated BTSCs were cultured in serum-free medium, the percentage and the time required for formation of brain tumor spheres (BTS) were observed.

**Results:**

**BTSCs **obtained by limiting dilution were all identified as CD133-positive by immunofluorescence. In serum-free medium, the proliferation of BTSCs in the ATRA group was observed significantly faster than that in the control group, but slower than that in the growth factor group and ATRA/growth factor group, and the size of the BTS in the ATRA group was smaller than that in the latter two groups(*P *< 0.01). In serum-containing medium, the expression percentages of CD133 and GFAP in the differentiated BTSCs were (2.29% ± 0.27%) and (75.60% ± 4.03%) respectively in the ATRA group, and (7.05% ± 0.49%) and (12.51% ± 0.77%) respectively in the control group. The differentiation rate of BTSCs in the ATRA group was significantly higher than that in the control group (*P *< 0.05), but there was still CD133 expressed in the ATRA group. The differentiated BTSCs could re-form BTSs in serum-free medium. The percentage of BTS formation in the ATRA group was(4.84% ± 0.32%), significantly lower than that in the control group (17.71% ± 0.78%) (*P *< 0.05), and the time required for BTS formation in the ATRA group was (10.07 ± 1.03)d, significantly longer than that in the control group (4.08 ± 0.35)d (*P *< 0.05).

**Conclusion:**

**ATRA **can promote the proliferation and induce the differentiation of BTSCs, but the differentiation is incomplete, terminal differentiation cannot be achieved and BTSs can be formed again.

## Introduction

All-trans retinoic acid (ATRA) is one of the strongest and most thoroughly studied differentiation inducers. It can induce the differentiation and apoptosis of a variety of tumor cells including glioma cells[1]. The concept of tumor stem cells suggests that the tumor stem cells are a cause of the formation, development and post-treatment relapse of tumors, as brain tumor stem cells (BTSCs) have a high potential of self-renewal and proliferation, which enables them to be resistant to chemo- and radiotherapies, so BTSCs must be eradicated in order to radically cure brain tumors. In this experiment, BTSCs are taken as the therapeutic target to study the effect of ATRA on the proliferation and differentiation of BTSCs, evaluating the antitumor activity of ATRA from a brand-new perspective.

## Materials and methods

### 1 Major reagents and instruments

(1) Major reagents: DMEM/F12 and B27 were purchased from Gibco(U.S.A). Epidermal growth factor (EGF) and basic fibroblast growth factor (bFGF) were purchased from PeproTech (U.S.A.). ATRA,3-(4,5-dimethylthiazol-2-yl)-2,5- diphenyltetrazolium bromide (MTT), fetal bovine serum (FBS), trypsin, Cy3-labeled sheep anti-rabbit IgG and diamidino-phenyl-indole (DAPI) were all purchased from Sigma (U.S.A). Rabbit anti-human CD133 antibody was purchased from Abcam (U.S.A). Rabbit anti-glial fibrillary acidic protein (GFAP) antibody and FITC-labeled goat anti-rabbit IgG were purchased from Boster (Wuhan, China).

(2) Major instruments: BB16 CO_2 _incubator and HF-safe-1200 purifying worktable (Heraeus and Lishen company, Germany). CKX41 inverted phase contrast microscope, BX51 fluorescence microscope and imaging system (Olympus, Japan). ELISA Reader 2010 (Anthos, Austria).

### 2 Experimental methods

(1) Isolation, culture and purification of BTSCs: The tissue samples were obtained from 3 surgical patients in Department of Neurosurgery, Anhui Provincial Hospital Affiliated to Anhui Medical University who had been diagnosed with glioblastoma during February-May, 2009. Fresh glioblastoma tissues without cystic degeneration, necrosis, calcification and electric coagulation were resected from the margin of tumor. By method in Ref[2], fresh glioblastoma tissues without cystic degeneration, necrosis, calcification and electric coagulation were resected from the margin of tumor, put in simplified serum-free medium (DMEM/F12, containing 2% B27, 20 g/L EGF and 20 g/L bFGF), and trimmed off necrotic tissues and residual blood vessels. After rinsing with serum-free medium for three times, the tissue masses were cut into pieces, disaggregated into single cell suspensions by using a Pasteur pipette (blowing repeatedly), and filtered with a sieve with the aperture of 74 m. The filtered single cell suspensions were stained with Trypan Blue. The living cells were counted, and primary culture was completed within 2 h, followed by inoculation in simplified serum-free medium (DMEM/F12, containing 2% B27, 20 μg/L EGF and 20 μg/L bFGF), and then culture at 37°C in 5% CO_2 _saturated humidity incubator. The medium was changed every 3~4 days. The cells were passaged by 1:2 subculture every 7 days and observed under the inverted phase contrast microscope. The cells were passaged three times. After the cell spheres became regularly shaped, they were dissociated into single cells with 0.25% trypsin + mechanical method, and inoculated into a 96-well plate at 1 living cell/well, with each well added with 100 μL simplified serum-free medium. The wells containing only one cell were labeled under the inverted microscope, and supplemented with 100 μL simplified serum-free medium for further culture. The formation of single cell colonies was recorded by dynamic observation. The cells were observed under the inverted microscope after culture for about one week, and the proliferated cells were collected and transferred into a culture flask for further culture and proliferation. The purified BTSCs after colony screening were used in the following experiments.

(2) Immunofluorescent identification of BTSCs: On the 5^th ^day of passage, BTSs that grew well were re-suspended in culture medium containing a small amount of serum (DMEM/F12 containing 10%FBS), and dropped onto a poly-L-lysine-coated coverslip. After standing still for about 4 h until the solution adhered to the coverslip, the coverslip was fixed in 4% paraformaldehyde for 30 min, blocked with normal goat serum for 20 min, incubated with rabbit anti-human CD133 antibody overnight at 4°C, and then incubated with Cy3-labeled sheep anti-rabbit IgG at 37°C for 60 min, followed by DAPI counterstaining of the nuclei and coverslipping with buffered glycerol. Following each step, the coverslip was rinsed with 0.01 mol/L PBS three times, each for 5 minutes. The coverslip was observed after mounting and pictures were taken.

(3) Assessment of the effect of ATRA on proliferation of BTSCs: The BTSCs were collected and divided into groups as described below, put into the corresponding culture medium, disaggregated into single cell suspensions by mechanical dissociation, and inoculated into a 96-well plate at the density of 1000 living cells/well, with 100 Ml in each well. According to the different treatments, the BTSCs were divided into: (1) control group: basic medium (DMEM/F12 with 2% B27) containing the same amount of anhydrous ethanol as in the ATRA group (the final concentration < 0.1%); (2) ATRA group: containing 1 μmol/L ATRA; (3) ATRA/growth factor group: containing 1 μmol/L ATRA, and 20 μg/L EGF and 20 μg/L bFGF; (4) growth factor group: containing 20 μg/L EGF and 20 μg/L bFGF. The cells in the plate were then cultured at 37°C in 5% CO_2 _saturated humidity incubator, each well was added with the corresponding medium 20 μL every other day, and the cell proliferation was observed under the inverted microscope every day. MTT assay was performed to evaluate the proliferation consecutively from the 1^st ^to the 9^th ^day of culture. Each well was added with 20 μL MTT solution (5 g/L), and the cells were cultured for 4 h, followed by 10 min centrifugation at 1000r/min. The supernatant in the wells was absorbed carefully and discarded. Each well was added with 150 μL DMSO. After shaking for 10 min to achieve dissolution and crystallization, the optical density value of each well was measured by ELISA at the wavelength of 570 nm. Six duplicate wells were set up for each group. The experiments were repeated 3 times, and the averages were obtained.

(4) Assessment of the effect of ATRA on differentiation of BTSCs: The collected BTSCs were adjusted to 2 × 10^5 ^living cells/mL using serum-containing medium (DMEM/F12 containing 10%FBS), and inoculated into a 6-well plate with PLL-coated coverslips, with 2 mL in each well. The cells were divided into two groups: (1) ATRA group: serum-containing medium added with ATRA with the final concentration of 1 μmol/L; (2) control group: serum-containing medium containing the same amount of anhydrous ethanol as in the ATRA group (the final concentration < 0.1%). The cells were cultured at 37°C in 5% CO_2 _saturated humidity incubator. The culture medium was changed every 3 days. The growth and differentiation of BTSCs were observed dynamically.

(5) Immunofluorescent detection of the differentiated BTSCs: The coverslips were taken out on the 10^th ^day of induction, fixed in 40 g/L paraformaldehyde for 30 min, blocked with normal goat serum for 20 min (those for GFAP staining were treated with 0.3%Triton X-100 for 20 min before serum blocking), incubated with anti-CD133 or anti-GFAP antibody overnight at 4°C, and then incubated at 37°C for 60 min with Cy3-labeled and FITC-labeled secondary antibodies respectively, followed by DAPI counterstaining of the nuclei and mounting with buffered glycerol. Following every step, the coverslips were rinsed with 0.01 mol/L PBS three times, each for 5 minutes. Randomly, 20 microscopic fields were selected on each coverslip and investigated under the fluorescence microscope to calculate the percentages of CD133 and GFAP positive cells among adherent cells. The calculation formula is: percentage of CD133 (or GFAP) positive cells = (CD133 (or GFAP) positive cells)/(DAPI positive cells)× 100%.

(6) Proliferation of the differentiated BTSCs: The adherent cells of the above two groups after 10 days of induction were digested with 0.25% trypsin, added with simplified serum-free medium, and inoculated into a 96-well plate at 5 living cells/well (density adjusted by limited dilution), with each well added with 100 μL simplified serum-free medium. The cells were then cultured at 37°C in 5% CO_2 _saturated humidity incubator. Each well was added with 20 μL simplified serum-free medium every other day, and the BTS formation was observed. The sphere formation and growth rate were observed at specified times every day, and the emergence of regularly-shaped BTSs (containing over 10 cells) was considered as positive result. The time required for BTS formation and the number of BTSs were recorded and used to calculate the percentage of BTS and the time for colony formation. The formed BTSs were dropped on PLL-coated coverslips to be dried for CD133 immunofluorescence staining as described previously.

### 3 Statistical analysis

All experimental data were expressed by mean ± standard deviation ($\bar{x}$ ± *s*). The software of SPSS version 16.0 was used for data analysis. An independent *t*-test was conducted for comparison between groups, and one-way ANOVA with Dunnett t test was used to compare the growth curves of different groups. *P *≤ 0.05 was considered statistically significant.

## Results

### 1 BTS formation from proliferation of a single BTSC

**Figure 1:**
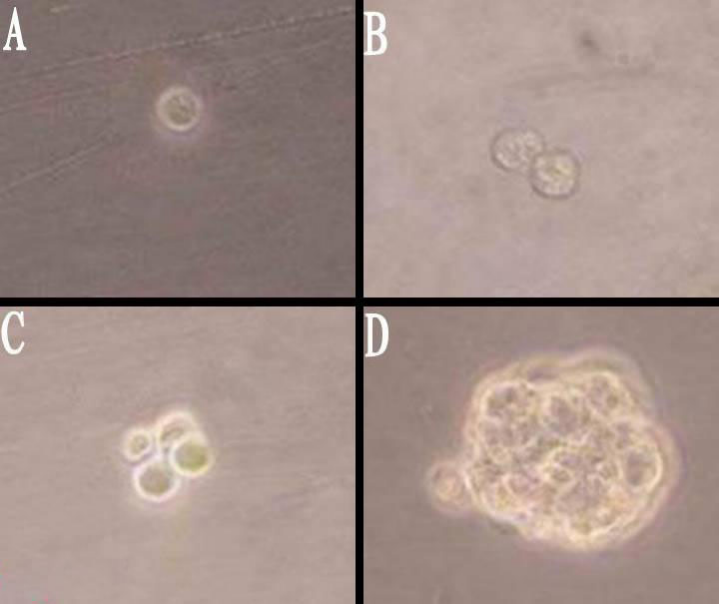
**BTS resulting from the proliferation of a single BTSC(Inverted phase-contrast microscope, × 400)**. 1A:an hour after inoculated. 1B: 12 hours after inoculated. 1C: 24 hours after inoculated. 1D: 3 days hours after inoculated.

**Figure 2:**
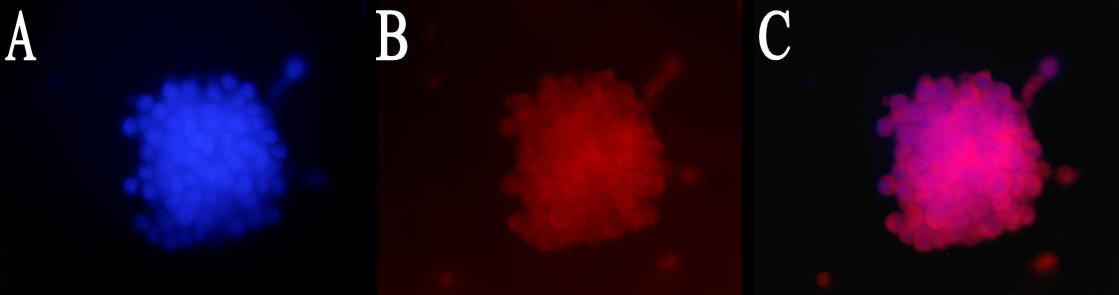
**Immunofluorescent identification of BTSCs for CD133 (Cy3, × 200)**. 2A: DAPI. 2B:CD133. 2C:Merge. It showed the cell spheres were CD133 positive.

The whole process of BTS formation from the proliferation of a single BTSC by limited dilution could be observed under the inverted microscope (Fig. 1). After 1-2 days of inoculation, it could be observed that the single cells splitted to form cell colonies consisting of 2~several cells. The cells in the colonies were round, with similar size. After 2~3 days, more cells formed colonies, and 4~5 days later, cell spheres composed of dozens to hundreds of cells were observed. The cell spheres were spherically shaped or elliptically shaped, with uniform structures and high transmittance. BTSCs are different from ordinary tumor cells due to their self-renewal and proliferation potential, and CD133 plays an important role in identifying whether BTSCs have the characteristics of stem cells, so cell spheres formed from the proliferation of a single cell were stained with CD133. It can be found that cell spheres were CD133 positive (Fig. 2), proving that the cultured cell spheres were composed of BTSCs with characteristics of stem cells. They could now be called BTS, which was the colonial sphere of a great number of sub-cell lines from the same cell, so the proportion of non-BTSCs was low, and the purity was high.

### 2 Proliferation of BTSCs promoted by ATRA

**Figure 3:**
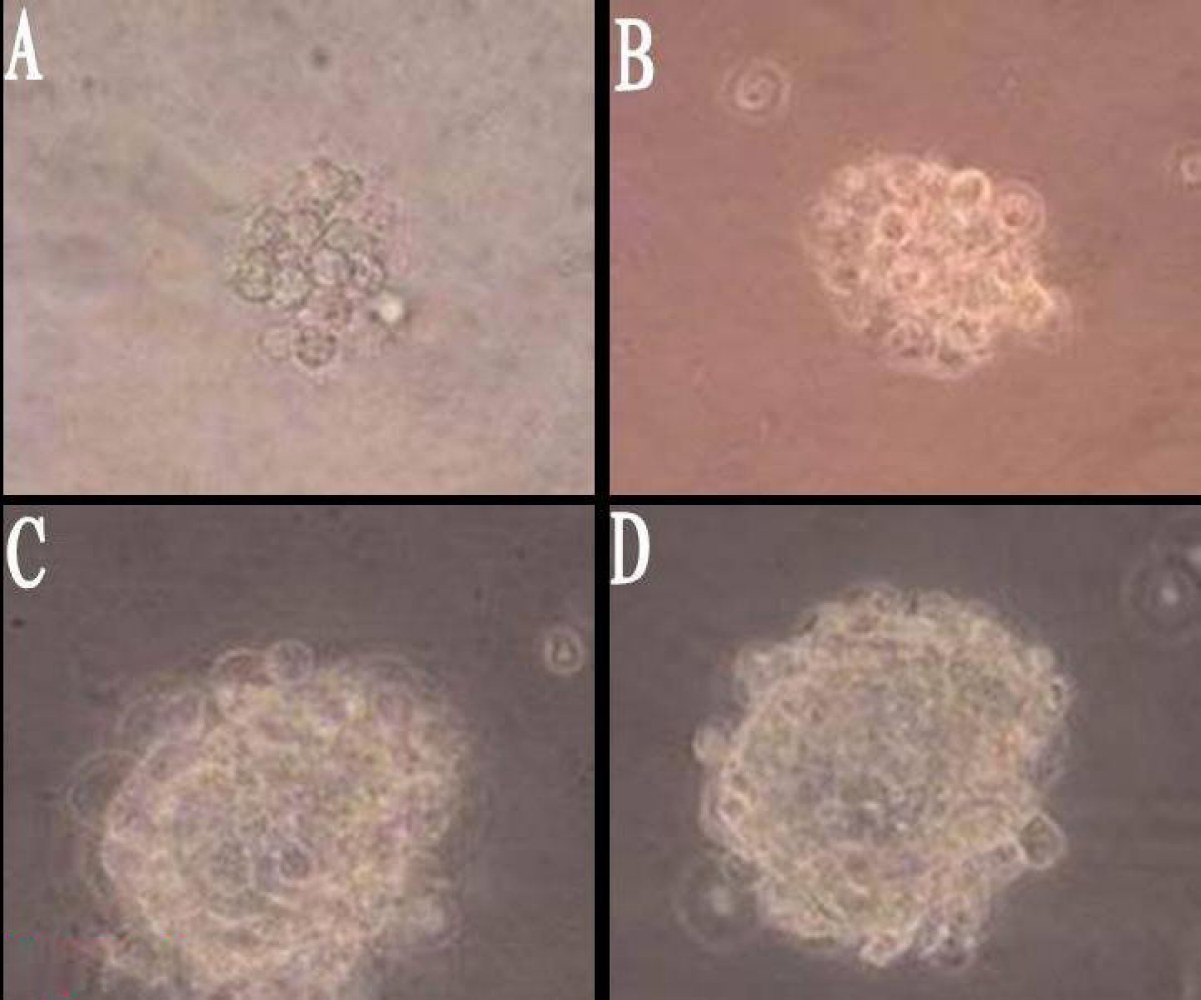
**The volume of the cell spheres formed in different group(Inverted phase-contrast microscope, × 400)**. 2A: the control group. 2B: the ATRA group. 2C: the ATRA/growth factor group. 2D: the growth factor group.

**Figure 4:**
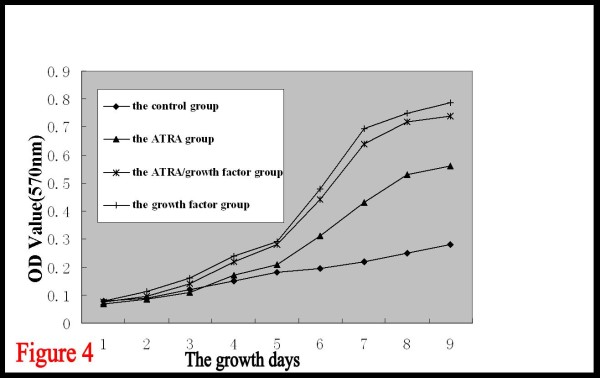
**Growth curves of BTSCs in different groups(the mean of optical density values measured for each group as the vertical axis, and the growth days as the horizontal axis)**. The results are shown as mean ± SD of four different experiment. Data of each day was analyzed by one-way ANOVA with Dunnett t test. The growth curves of the ATRA group, ATRA/growth factor group and growth factor group rise faster than that of the control group(P < 0.01). While there were no statistically significant between the ATRA/growth factor group and growth factor group(P > 0.05).

BTSCs in the growth factor group began to proliferate after 1~2 days of culture, forming cell spheres composed of 10~20 cells. The cells exhibited rapid suspended growth thereafter, and the cell spheres gradually got larger. Seven days later, dozens to hundreds of cells aggregated to form sphere-like structures with distinct boundary and high refraction. Cell proliferation occurred after 2~3 days of culture in the ATRA/growth factor group. The cell growth in this group was almost the same as in the growth factor group, but the number and volume of the cell spheres formed were slightly smaller than those in the growth factor group. Cell proliferation also occurred after 2~3 days in the ATRA group, with the cell spheres exhibiting suspended growth, but only cell masses consisting of dozens of cells were observed during the whole process. The volume of the cell spheres was larger than that in the control group, but obviously smaller than that in the growth factor group and the ATRA/growth factor group. The cell proliferation in the control group was relatively slower, and the formed colonies were smaller, merely consisting of a dozen cells (Fig. 3). No obvious adherent differentiation was observed in any group. With the mean of optical density values measured for each group as the vertical axis, and the growth days as the horizontal axis, the growth curves of BTSCs for different groups were plotted (Fig. 4) to compare the cell proliferation rates of the four groups. It can be observed that, on the 1^st^-3^rd ^day, the growth curves of all the four groups rise slowly, with an insignificant difference in the cell proliferation rate. From the 3^rd ^day, the cell proliferation obviously become more rapid, and the growth curves of the four groups begin to separate from each other. The curve is steep during the 5^th^~7^th ^days, indicating the peak of proliferation. Cell proliferation is slowest in the control group, obviously faster in the ATRA group, and fastest in the growth factor group, and the proliferation rate of the ATRA/growth factor group is slightly lower than that of the growth factor group, but significantly higher than that of the ATRA group. It is indicated that ATRA had a promotive effect on the proliferation of suspended BTSCs, but had no obvious synergistic or antagonistic effect with the growth factor.

### 3 Differentiation of BTSCs promoted by ATRA

**Figure 5:**
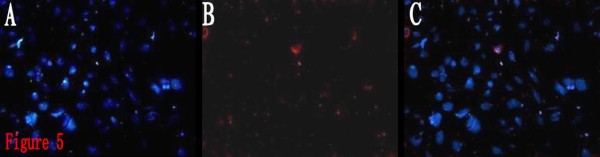
**Immunofluorescence staining of differentiated BTSCs for CD133 (Cy3, × 200)**. 5A: DAPI. 5B:CD133. 5C:Merge. It showed the CD133 expression of differentiated BTSCs induced by ATRA did not disappear.

**Figure 6:**
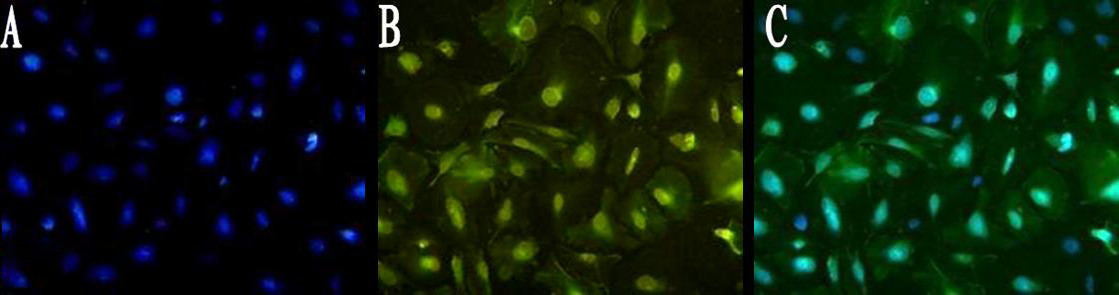
**Immunofluorescence staining of differentiated BTSCs for GFAP (FITC, × 200)**. 6A: DAPI. 6B:GFAP. 6C:Merge. It showed the differentiated BTSCs induced by ATRA were GFAP positive.

It can be observed under ordinary light microscope that, after 6 h, BTSCs of both groups were adherent, and generated small protuberances toward outside. With the prolonging of the protuberances, the protuberances of the adjacent cells formed a netlike connection. The BTSCs grew larger, becoming different in size and shape, exhibiting the shapes of polygon, spindle and roundness, and being transparent under microscope, with high refraction. DAPI staining showed that the nuclei had different sizes and shapes, with significant atypia. There was no obvious increase in the adherent cells, indicating that proliferation of BTSCs was inhibited in the serum-containing medium, and cell differentiation was dominant. CD133 and GFAP immunofluorescence detection of the expression percentages after 10 days of induction by ATRA showed that CD133 expression did not disappear in both groups, indicating that BTSCs did not achieve terminal differentiation, and had the characteristics of being differentiated incompletely. But compared to the control group, the CD133 expression in the ATRA group was lower, and the GFAP expression was higher, the differences being significant (*P *< 0.05) (Fig. 5, 6, Table 1). It is indicated that ATRA can induce the differentiation of BTSCs, however, can not help the BTSCs to achieve terminal differentiation, but instead can promote the proliferation and self-renewal of BTSCs.

**Table 1:** The expressions of the markers, percentage and time of BTS formation in the differentiated BTSCs(n = 3, *Mean *± *SD*)

Group	CD133 (%)	GFAP(%)	the percentage of BTS	the time of formation
control group	7.05 ± 0.49	12.51 ± 0.77	17.71 ± 0.78	4.08 ± 0.35
ATRA group	2.29 ± 0.27	75.60 ± 4.03	4.84 ± 0.32	10.07 ± 1.03

*T *value	14.737	26.634	26.440	9.537
*P *value	0.000	0.000	0.000	0.001

### 4 Reduction of proliferation of the differentiated BTSCs by ATRA

**Figure 7:**
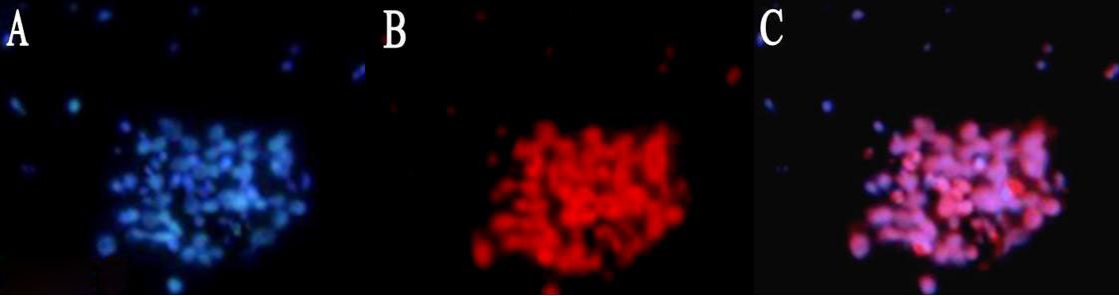
**Immunofluorescence staining of BTS generated from differentiated BTSCs for CD133(Cy3, × 400)**. 7A: DAPI. 7B:CD133. 7C:Merge. It showed the BTS obtained from differentiated BTSCs were CD133 positive.

Within 24 hours after the differentiated BTSCs were transferred into the simplified serum-free medium, a majority of cells became adherent and generated protuberances, with a minority suspending in the medium. After 2 days of culture, some of the suspended cells in the control group proliferated to form cell masses. After 3~6 days, more cells aggregated to form masses, and a great number of suspended cell masses emerged one after another, which consisted of a dozen cells at first, and gradually grew larger with the lapse of time and became sphere-shaped, with each sphere composed of 100~200 cells of similar size. In the ATRA group, suspended cell masses began to appear on the 9^th ^day, and gradually increased in number during the following 3~4 days, but the formed spheres had smaller diameters and slower growth rate. Table 1 shows that the percentage of BTS formation in the ATRA group was (4.84% ± 0.32%), significantly lower than that in the control group (17.71% ± 0.78%) (*P *< 0.05), and the time required for BTS formation in the ATRA group was (10.07 ± 1.03)d, significantly longer than that in the control group (4.08 ± 0.35)d (*P *< 0.05). The BTSs obtained from differentiated BTSCs were CD133 positive (Fig. 7), indicating that stem cell phenotype was restored again. Accordingly, the differentiated BTSCs induced by ATRA did not accomplish terminal differentiation and lose the proliferation capability. ATRA can induce the differentiated BTSCs into more mature ones, but the induction is not thorough and complete, and terminal differentiation cannot be achieved.

## Discussions

Ever since Singh et al discovered BTSCs for the first time in 2003[2], many scholars have confirmed that BTSCs exist in the brain tumor tissue and its cell lines, and possess the potential of self-renewal, unlimited proliferation, multilineage parent differentiation and high tumorigenicity[3-6]. In 2004, Galli et al and Singh et al proposed a new tumorigenesis model, believing that BTSCs were the initiating cells of tumor formation[4,5]. These BTSCs proliferated and differentiated following the same symmetric and asymmetric division rule as neural stem cells, namely, accomplishing self-renewal and proliferation by symmetric division, and producing relatively mature progeny cells by asymmetric division which can be differentiated into more mature tumor cells.

Induction of differentiation of glioma cells into benign ones has been one of the research focuses of glioma therapy in recent years. The application of differentiation inducers can increase the differentiation of the tumor cells and inhibit proliferation. ATRA, as a classic differentiation inducer, has achieved a very good curative effect in clinical treatment of hematological neoplasms and lymphoma. In vitro study has indicated that ATRA can induce the differentiation and apoptosis of a variety of glioma cells[7]. Many researches have confirmed that BTSCs are able to self renew and proliferate continuously when cultured in serum-free medium containing growth factor, retaining the inherent feature of stem cells, but differentiate into tumor cells with the shape and molecular phenotype resembling the parental tumor under serum-containing conditions[2-6]. This study has used BTSCs as the therapeutic target to investigate the effect of ATRA on the proliferation and differentiation of BTSCs both in the serum-free and serum-containing mediums.

BTSCs with a high purity must be obtained first in order to do research on BTSCs. Currently, the method of immunomagnetic bead separation is often used to obtain CD133-positive BTSCs at home and abroad, which is expensive and has a primary isolation efficiency of merely 46.9%~79.8%[3]. Che Xiaoming et al achieved similar outcomes by colony selection with the use of limited dilution, and harvested about 82% cells that have the proliferation capacity[2]. We obtained highly purified BTSCs by their method.

As is known to all, EGF and bFGF, as powerful promoters of cell division, are essential key components in stem cell culture medium, and enable stem cells to proliferate continuously. Through MTT experiment, we have found that ATRA alone can promote the proliferation of BTSCs, but the promoting effect is weaker than EGF+bFGF, and there is no obvious synergistic or antagonistic effect between ATRA and EGF+bFGF. Previous researches have showed that ATRA can inhibit the proliferation of ordinary glioma cells cultured in serum-containing medium, promoting apoptosis of the glioma cells. We have observed that BTSCs in the control group grew as suspended spheres when cultured in the medium without serum and growth factors. Similar to the control group, BTSCs in the ATRA group were not adherent, but the formed spheres were larger and the proliferation was more rapid, indicating that ATRA did not induce the differentiation of the suspended BTSCs, but promote the proliferation of BTSCs. The reason may be as mentioned below. In the serum-free medium, BTSCs can achieve continuous self renewal and proliferation through symmetric division, retaining the stem cell characteristics; and in the serum-containing medium, because of the influence of certain substance in the serum, BTSCs can retain their existence through asymmetric division, and produce a great number of comparatively mature progeny cells, which differentiate into ordinary tumor cells ultimately, so there is only a small percentage of BTSCs in the whole cell population. The targets of ATRA's effect of differentiation induction are cells in the process of differentiation. For BTSCs in the stem cell state, ATRA has a promoting effect on their proliferation. So ATRA exerts opposite effects on BTSCs at different stages of differentiation, the mechanism of which needs further clarification. Clinical trials of differentiation of brain glioma cells induced by ATRA showed that the differentiation effect of ATRA alone was weak, with insignificant curative efficacy[8,9]. We speculate that the application of ATRA alone can induce the differentiation and apoptosis of most ordinary glioma cells, but promote the proliferation of a minority of BTSCs that does not experience differentiation, that is to say, the "seeds" resulting in the formation, development and relapse of tumors do not decrease but increase, which may be exactly the major reason for the poor therapeutic effect.

Research of Singh et al revealed that only CD133 positive cells had the stem cell characteristics of self-renewal, unlimited proliferation and multilineage parent differentiation[3]. These days, CD133 has been recognized as the marker to isolate and identify BTSCs. And the astrocyte marker GFAP is one of the major markers for the differentiated progeny of BTSCs, and GFAP expression is also increased after glioma cells differentiate into mature ones. Engelhard et al found that the loss of GFAP expression could promote the malignant phenotype of cells and accelerate the development of glioma, whereas the up-regulation of GFAP expression could promote the differentiation of glioma, reducing the malignancy[10]. Toda et al [11], after tranfecting rat C6 glioma cell line with GFAP cDNA, found that the cell growth was inhibited and GFAP expression increased, showing a differentiation trend, and believed that GFAP gene could inhibit tumors. Besides, some negative regulator genes of cell cycles can also induce differentiation through GFAP gene[11]. For instance, transfection of P21WAF1/CIP1 gene can enhance the GFAP expression, thus enabling the tumor cells to achieve terminal differentiation [12]. Accordingly, we used CD133 and GFAP to examine the induction effect of ATRA on the differentiation of BTSCs from the level of molecular biology. BTSCs differentiated in serum-containing medium, and the differentiated BTSCs expressed more GFAP and less CD133 with the addition of ATRA, and meanwhile the proliferation ability was reduced. It can be believed that ATRA induces the differentiation of BTSCs into more mature ones, and prevents the differentiated BTSCs from differentiating to form more BTS, reducing the differentiation capacity of BTSCs to a certain extent. Therefore, ATRA has a dual effect on BTSCs: (1) multiplying BTSCs by promoting proliferation and self renewal; (2) inducing differentiation of the differentiated BTSCs into more mature ones through indirectly up-regulating the GFAP expression.

It has been found in this study that CD133 expression did not disappear after differentiation of BTSCs induced by ATRA in serum-containing medium. The differentiated BTSCs were still able to differentiate and proliferate to form BTSs after being inoculated into serum-free medium that was added with growth factors. However, after differentiation of NSCs, though cells with the NSC phenotype still exist among the differentiated cells, they don't have the ability of re-forming neurospheres[13]. These abnormal phenomena indicate that ATRA-induced differentiation therapy fails to achieve terminal differentiation of BTSCs and enable them to lose the proliferation ability, and the differentiated BTSCs can restore the characteristics of stem cells under certain conditions, which may be the major reason for the poor effect of this therapy. With the deepening of the investigation into BTSCs, the key to achieve breakthrough in this area is to further reveal the molecular mechanism of the proliferation and differentiation of BTSCs and develop the differentiation inducer specific for BTSCs.

## Competing interests

The authors declare that they have no competing interests.

## Authors' contributions

NCS, LMW and NYF designed the experiments. NCS and LMW carried out most of experiments and drafted the manuscript. CJM and MJM carried out the immunoassays. LJ and FXM participated in statistical analysis and interpretation of data. All authors read and approved the final manuscript.
